# A microarray-based approach to evaluate the functional significance of protein-binding motifs

**DOI:** 10.1007/s00216-016-9382-6

**Published:** 2016-02-18

**Authors:** Michael D. Sinzinger, Yi-Da Chung, Merel J. W. Adjobo-Hermans, Roland Brock

**Affiliations:** Department of Biochemistry, Radboud Institute for Molecular Life Sciences, Radboud University Medical Center, Geert Grooteplein 28, 6525 GA Nijmegen, The Netherlands

**Keywords:** Peptide microarray, Dissociation constant, EC50, T cell signalling, Cheng-Prusoff equation, Protein interaction

## Abstract

Intracellular proteins comprise numerous peptide motifs that interact with protein-binding domains. However, using sequence information alone, the identification of functionally relevant interaction motifs remains a challenge. Here, we present a microarray-based approach for the evaluation of peptides as protein-binding motifs. To this end, peptides corresponding to protein interaction motifs were spotted as a microarray. First, peptides were titrated with a pan-specific binder and the apparent *K*_d_ value of this binder for each peptide was determined. For phosphotyrosine-containing peptides, an anti-phosphotyrosine antibody was employed. Then, in the presence of the pan-specific binder, arrays were competitively titrated with cell lysate and competition constants were determined. Using the Cheng-Prusoff equation, binding constants for the pan-specific binder and inhibition constants for the lysates were converted into affinity constants for the lysate. We experimentally validate this method using a phosphotyrosine-binding SH2 domain as a further reference. Furthermore, strong binders correlated with binding motifs engaging in numerous interactions as predicted by Scansite. This method provides a highly parallel and robust approach to identify peptides corresponding to interaction motifs with strong binding capacity for proteins in the cell lysate.

**Electronic supplementary material:**

The online version of this article (doi:10.1007/s00216-016-9382-6) contains supplementary material, which is available to authorized users.

**Graphical Abstract:**
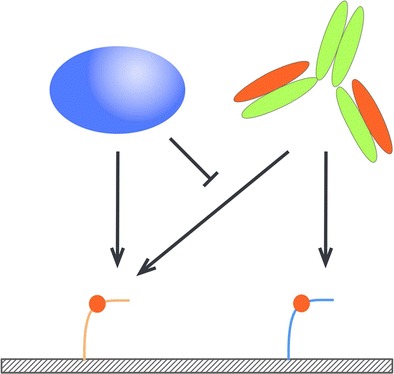
Using an antibody as a pan-specific binder the capacity of interaction motifs to bind to proteins from cell lysates can be probed. Competition of the antibody is observed for only those peptides to which a lysate protein binds

## Introduction

In the analysis of biomolecular interactions, microarray-based approaches have greatly expanded the information content that can be obtained in one single experiment. The interaction of one target molecule with numerous immobilised capture molecules can be probed in a highly parallel fashion. Besides DNA microarrays [1], microarrays are used to study protein interactions with antibodies [2], proteins [3] or peptides [4] as capture molecules immobilised on a functionalised surface. In addition to providing qualitative data on interaction preferences, microarrays can also be used for obtaining quantitative data on molecular interactions [5, 6].

In cellular signal transduction, a major part of protein-protein interactions are realised by modular domains [7–9]. Interaction networks have been inferred by a compilation of binding patterns of probe molecules that were individually incubated on microarrays of capture molecules. In these studies, capture molecules comprised of all members of a given type of interaction domain and probe molecules were peptide motifs [10, 11].

However, to characterise the functional significance of an interaction motif in engaging in molecular interactions in comparison to related motifs, this compilation of binding profiles is inefficient. Instead, a method is needed that provides direct access to the affinity of interaction motifs towards the cellular proteome. As we have shown before, instead of using purified binders, crude cell lysates can also be used as a source of target proteins, enabling for example the detection of signalling-dependent changes in molecular interactions [12, 13] and the effect of endogenous competitors on binding profiles [14].

In this study, we set out to further extend these concepts to provide information on the general affinity of interaction motifs immobilised on the microarrays towards the cellular proteome. Phosphotyrosine-containing peptides, interacting with SH2 domains, were selected to validate the approach. In a first step, microarrays were titrated with an α-phosphotyrosine antibody serving as a pan-specific binder. In a second step, binding of the antibody was competed out by incubation with cell lysate. Using the apparent dissociation constants for the pan-specific binder as derived from the binding isotherms [15, 16], EC_50_ values for the lysate were converted into binding constants for the proteome using the Cheng-Prusoff equation [17]. We further validated the approach for a protein domain as a reference binder, extending the applicability of the method to interactions for which a pan-specific antibody is not available. The prediction of functional significance showed a strong positive correlation with the number of interactors as predicted by Scansite [18]. In the functional characterisation of interaction motifs, this approach constitutes a highly valuable alternative to the compilation of binding patterns.

## Material and methods

### Generation of peptide microarrays

Peptides that correspond to known interaction motifs involved in the formation of protein complexes in T cell signalling were purchased from EMC microcollections (Tübingen, Germany). All peptides carried an extra cysteine residue and a free N-terminus for immobilisation on the microarray substrates and a C-terminal amidation (see Electronic Supplementary Material (ESM), Table S1).

For generation of microarrays, peptide stock solutions (3 mM) in DMF were diluted 30 times with phosphate buffer (100 mM, pH 8) supplemented with 0.006 % (*v*/*v*) Triton X-100. *N*-Hydroxysuccinimide-preactivated slides served as substrates for immobilisation (Nexterion Slide H, Schott Nexterion/Peq-Lab, Erlangen, Germany) using a Nano-Plotter NP2.0 (GeSIM, Großerkmannshof, Germany), which employs piezo-driven pipetting tips to dispense peptide solutions. Microarray slides comprised 16 subarrays of doublets of each peptide. For each peptide, a volume of 1.2 nl was dispensed at a centre-to-centre spacing of 500 μm. During printing, temperature was maintained at 15 °C and air humidity at 65 %.

### Preparation of cell lysates

Jurkat T cell leukaemia cells were maintained in RPMI 1640 medium (PAN-Biotech, Aisenbach, Germany) supplemented with 10 % foetal calf serum at 37 °C in 5 % CO_2_. For the preparation of lysates, cells were resuspended in lysis buffer (1 % Triton X-100, 50 mM *n*-octyl-d-glucopyranoside (Fluka, Taufkirchen, Germany), 20 mM Tris, 1 mM EDTA, 150 mM NaCl, 1 mM Na_3_VO_4_, pH 7.5, and complete protease inhibitor cocktail (Roche Applied Science, Mannheim, Germany)). Incubation on ice for 1 h was followed by centrifugation at 20,000×*g* at 4 °C for 15 min to remove cell debris. Transmembrane proteins were extracted from lipid rafts by using *N*-octyl-d-glucopyranoside. Protein concentrations were measured by Bradford Assay.

### Expression and purification of recombinant GRB2-SH2

The PCR product of the SH2 domain of GRB2 (GRB2-SH2) was cloned into the pET160/GW/D-TOPO vector (Invitrogen, Breda, The Netherlands), which contains a region encoding for a His-tag. The plasmid was used to perform a transformation into One Shot TOP10 competent cells (Invitrogen). Bacteria were lysed in CellLytic B buffer (Sigma-Aldrich, Steinheim, Germany) and 1 min of sonication was used to lyse the transformed bacteria. Pellets were created by spinning the lysates twice for 10 min at 20,000×*g*, 4 °C. Protein purification from the protein-containing supernatant was performed with HIS-Select Cartridges H 8286 (Sigma-Aldrich, Steinheim, Germany). This step was followed by a dialysis against phosphate-buffered saline (PBS) with the addition of 1 mM mercaptoethanol for 3 days at 4 °C while stirring in a dialysis cassette (Slide-A-Lyzer, Pierce, Rockford, USA). The purified protein solution was further concentrated by an Amicon Ultra—15 and Ultracel—5 k centrifugation tube (Millipore, Schwalbach, Germany). The concentration of expressed protein was determined by photometric measurements at OD_280_. The concentration of GRB2-SH2 was determined in Western blots by quantitatively analysing obtained bands of purified GRB2-SH2 in an Odyssey Infrared Imaging System (Li-Cor Biosciences, Bad Homburg, Germany) in comparison with a bovine serum albumin (BSA) standard.

### Microarray experiments

Sixteen incubation chambers with identical subarrays consisting of peptides derived from binding motifs of signalling proteins (see ESM Table S1) were generated by attaching a clip-on frame to the microarray substrate (ProPlate Multiarray System, Grace Biolabs, Molecular Probes, Eugene, OR, USA). The typical incubation volume for the subarrays was 50 μl. Microarrays were either incubated with an α-pY antibody (α-pTyr 100, 9411, Cell Signalling, Frankfurt am Main, Germany) at the indicated concentrations or co-incubated with cell lysate at the indicated concentrations for 3 h at 4 °C. Additionally, in another set of experiments, recombinant GRB2-SH2 was incubated on microarrays alone or co-incubated with cell lysate employing an α-penta HIS antibody (34600, Qiagen, Hilden, Germany) for the detection of His-tagged GRB2-SH2.

After incubation, microarrays were washed three times with washing buffer (PBS, 0.05 % (*w*/*v*) BSA, 0.05 % (*w*/*v*) Tween-20). For the detection of signals, microarrays were incubated with a secondary α-mouse antibody conjugated with Alexa633 (A-21050, Invitrogen) at a concentration of 1 μg/ml for 15 min at RT followed by a last washing step with washing buffer. Microarrays were dried with nitrogen and scanned using a ProScanArray (PerkinElmer Life Sciences, Waltham, Massachusetts), and the data were analysed using ArrayPro Analyzer software (Media Cybernetics, Silver Spring, USA) as described before [13, 14]. Final data represented the mean signal intensities corrected for the median of a ring-shaped local background surrounding the respective peptide spot. Titrations with α-pY antibody and GRB2-SH2 were performed to calculate dissociation constants (see below).

### Quantitative analyses of molecular interactions

The resulting titration curves were fitted with Origin (OriginLab Corporation, v.6.1057, Northampton, USA) using a binding isotherm for a bimolecular interaction (Eq. 1):

1 $$ {F}_{\mathrm{abs}}=\frac{F_{\max}\left[\mathrm{prot}\right]}{K_d+\left[\mathrm{prot}\right]} $$

*F*_abs_ represents the intensity of a microarray spot after background correction and [prot] the concentration of the titrated binder. *K*_d_ and *F*_max_ were obtained from the fits.

For determination of EC_50_ values for competition with cell lysate, curves were fitted according to

2 $$ y={y}_0+A{e}^{-x/t} $$

with *y*_0_ as the signal intensity of the α-pY antibody or the recombinant GRB2-SH2 domain without cell lysate, *A* the amplitude, *x* the protein concentration in lysate, and *t* the decay constant. EC_50_ values were calculated with

3 $$ {\mathrm{EC}}_{50}=- \ln 0.5t $$

With knowledge of the EC_50_ values of the antibody or protein domain for the individual peptides, *K*_d_ values for the lysate proteins were derived using the Cheng-Prusoff equation [17]

4 $$ {K}_{\mathrm{d},\kern0.5em \mathrm{lysate}\kern0.5em \mathrm{protein}}=\frac{{\mathrm{EC}}_{50}}{1+\frac{\left[\alpha -pY\right]}{K_{\mathrm{d},\kern0.5em \alpha -pY}}} $$

This dissociation constant reflects the affinity of the entirety of all proteins in the lysate towards a binding motif on the microarrays.

## Results

### Determination of *K*_d_ and EC_50_ for an anti-phosphotyrosine antibody

**Fig. 1:**
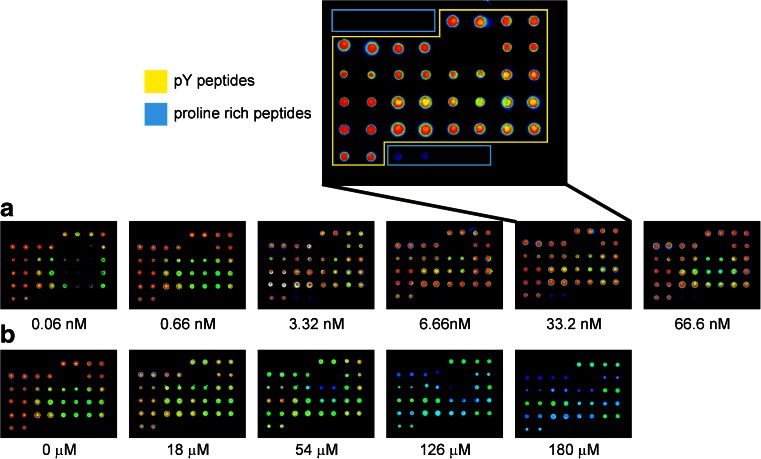
Titration and competition of α-pY antibody binding to peptide microarrays. **a** Titration of α-pY. Concentrations refer to the concentrations of the α-pY antibody. Binding was detected by indirect immunofluorescence. **b** Competition of α-pY binding through addition of increasing concentrations of cell lysate. The indicated ‘concentrations’ refer to the total concentration of protein in cell lysates determined by Bradford Assay. α-pY was used in a fixed concentration of 0.33 nM

To obtain general information on the activity of a peptide motif as a binder for the total cellular proteome, a competitive approach was selected. We focused our analysis on peptides containing phosphotyrosine motifs (ESM Table S1) that bind to protein domains such as the SH2 domain. An anti-phosphotyrosine-directed antibody was chosen as a pan-specific binder that could be detected by indirect immunofluorescence. Binding of cellular proteins could then be detected by competition of antibody binding (Fig. 1).

**Fig. 2:**
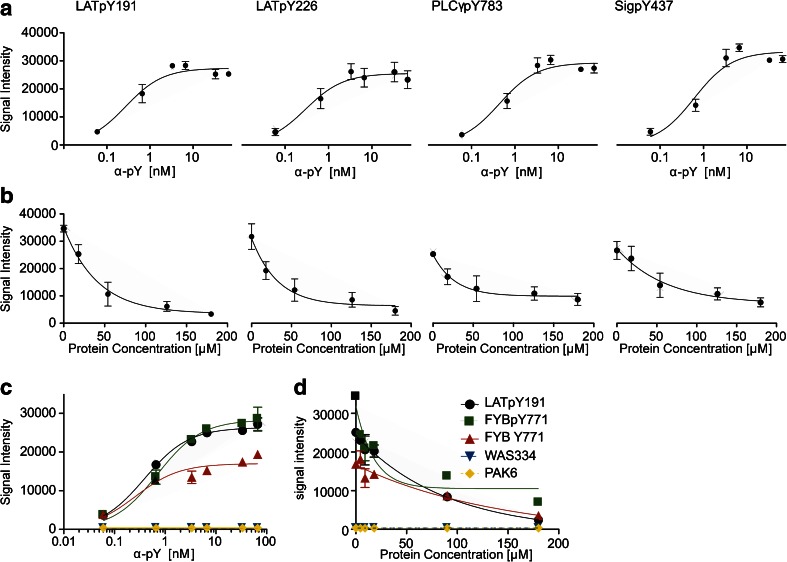
Examples for binding curves for titration and competition of α-pY antibodies on the peptide microarrays. **a** Titration curves on four different phosphotyrosine peptides LATpY191, LATpY226, PLCγpY783 and SigpY437. Depicted are means and standard errors of six independent experiments. **b** Competitions were performed with a fixed concentration of 0.33 nM α-pY and increasing concentrations of cell lysate which were converted into molar protein concentrations assuming an average molecular weight corresponding to the one of BSA. The displayed competition curves are for the peptides from the respective titration curve above (**a**) and represent means and standard errors of four independent experiments. **c** Control experiments for the specificity of the α-pY antibody on the microarrays. Titration curves for the α-pY antibody on two phosphorylated peptides, one non-phosphorylated peptide and two proline-rich peptides are shown. **d** Control experiments for the specificity of binders in cell lysate. Cell lysates of Jurkat cells in different concentrations per subarray were co-incubated with the α-pY antibodies to check for the ability of proteins in the lysate to compete with antibody binding. Results for two phosphorylated peptides, one non-phosphorylated peptide and two proline-rich peptides are depicted. **c**, **d** Show means of relative signal intensities and refer to two independent experiments; the *error bars* indicate standard errors

To render this approach quantitative, first the affinities of the α-pY antibody towards the phosphotyrosine motifs of the microarrays were determined. Microarrays were titrated in six independent experiments with the antibody at concentrations ranging from 0.06 to 6.66 nM, which showed a concentration-dependent binding to the array towards saturation. This was observed for all of the phosphotyrosine peptides. The titration curves were fitted with a binding isotherm for a bimolecular interaction (Eq. 1, Fig. 2a, Table 1). In general, a coefficient of determination (*R*^2^) of 0.8 was used as a cutoff in order to score a peptide as a binder. For all phosphotyrosine (pY)-containing peptides, titration curves with an overall average coefficient of determination (*R*^2^) of 0.85 and average *K*_d_ values for the single peptides ranging from 0.28 to 1.32 nM could be obtained. Regarding the poly-proline motif (PP) containing peptides present on the microarray, only on the peptide GAB2-509 in five experiments, a titration curve with an *R*^2^ > 0.8 could be generated.

**Table 1:** Dissociation constants and EC_50_ values for binding of proteins to phosphotyrosine peptides. EC_50_ values were determined by competition of α-pY with cell lysate. Error ranges represent standard deviations. As EC_50_ values were calculated from averaged competition curves, standard deviations for *K*_d_ values of the approach using GRB2-SH2 are not available

Peptide	*K*_d_ α-pY [nM]	EC_50_ [μM]	*K*_d_ lysate proteins (α-pY) [μM]	*K*_d_ lysate proteins (GRB2-SH2) [μM]
SigpY437	0.66 ± 0.46	44 ± 40	22 ± 20	0.1
LATpY132	0.41 ± 0.38	29 ± 34	11 ± 13	1.5
CD3ζpY72/83	0.69 ± 0.61	50 ± 53	25 ± 27	1.3
PLCγpY783	0.55 ± 0.33	16 ± 5	7 ± 2	1.0
ZAPpY296	1.32 ± 0.59	43 ± 45	27 ± 28	3.3
ZAPpY319	0.60 ± 0.46	54 ± 68	26 ± 32	2.0
LATpY226	0.44 ± 0.27	37 ± 27	15 ± 11	2.2
LATpY191	0.35 ± 0.28	47 ± 44	16 ± 15	4.9
SHP1pY564	0.89 ± 0.57	30 ± 17	17 ± 10	4.4
FYBpY595	0.77 ± 0.28	41 ± 31	17 ± 19	–
FYBpY625	0.46 ± 0.15	50 ± 26	17 ± 13	–
FYBpY651	0.28 ± 0.09	54 ± 28	12 ± 9	–
FYBpY771	0.50 ± 0.23	12 ± 3	5 ± 1	–

– = not determined

Next, the ability of cellular proteins to compete for antibody binding was assessed (Fig. 2b). EC_50_ values for inhibition of α-pY antibody binding were acquired by incubation of the α-phosphotyrosine (α-pY) antibody in a fixed concentration (0.66 nM) with lysates of Jurkat cells in concentrations of 18, 54, 126 and 180 μM of the total lysate protein (four independent experiments). The protein content in the cell lysates was determined by Bradford Assay and converted into a molar protein concentration assuming the molecular weight of BSA (*M*_BSA_ = 66,382 g/mol). This is certainly an arbitrary choice but gave us the possibility to place the titration curves in the context of molar affinities. A total loss of signal of the α-pY antibody was obtained for only a fraction (e.g. LATpY191) of peptides, validating the strength of binding of the α-pY to the pY motifs. All competition experiments were fitted using Eq. 2, and EC_50_ values were calculated according to Eq. 3. Average EC_50_ values ranged from 12.28 to 54.07 μM. Both for the titration and the competition experiments, raw data were averaged without normalisation (Fig. 2a, b) as maximum signal intensities between single microarrays were in the same range.

In addition to the phosphotyrosine peptides, a set of proline-rich peptides (ESM Table S1) and, in an additional experimental series, one non-phosphorylated peptide (FYB Y771) were also included on the arrays and served as internal controls for the specificity of the competition for phosphotyrosine-containing motifs. The α-pY antibody showed binding on the non-phosphorylated FYB Y771 peptide (see Fig. 2c); however, the maximum signal intensities on FYB Y771 were only half of the ones on FYBpY771 suggesting a high off-rate of the α-pY antibody on this peptide. In direct comparison of the phosphorylated variant of the FYB peptide with the non-phosphorylated one, in competition experiments with cell lysate, a difference in the EC_50_ values of almost 1 order of magnitude could be observed (12.24 μM for FYBpY771 vs. 100.3 μM for FYB Y771). This demonstrates that the phosphotyrosine-containing peptide probed specifically for binding of proteins from the cell lysate as the α-pY antibody is competed off more efficiently on the pY peptide compared to the non-phosphorylated peptide. That a titration curve could be detected at all, we consider a coincidence resulting from the residual binding capacity of the antibody on this peptide.

For the two proline-rich peptides WAS334 and PAK6 depicted in Fig. 2c, no titration curves could be generated. This is in line with the signals shown in the magnification in Fig. 1a.

These results are further confirmed by earlier experiments, in which competition experiment were performed with peptides in solution corresponding to arrayed peptides and a concentration-dependent decrease of signal intensities for proteins binding to the arrayed peptides could be detected [13].

### Determination of the general affinity of cellular proteins towards pY motifs

**Fig. 3:**
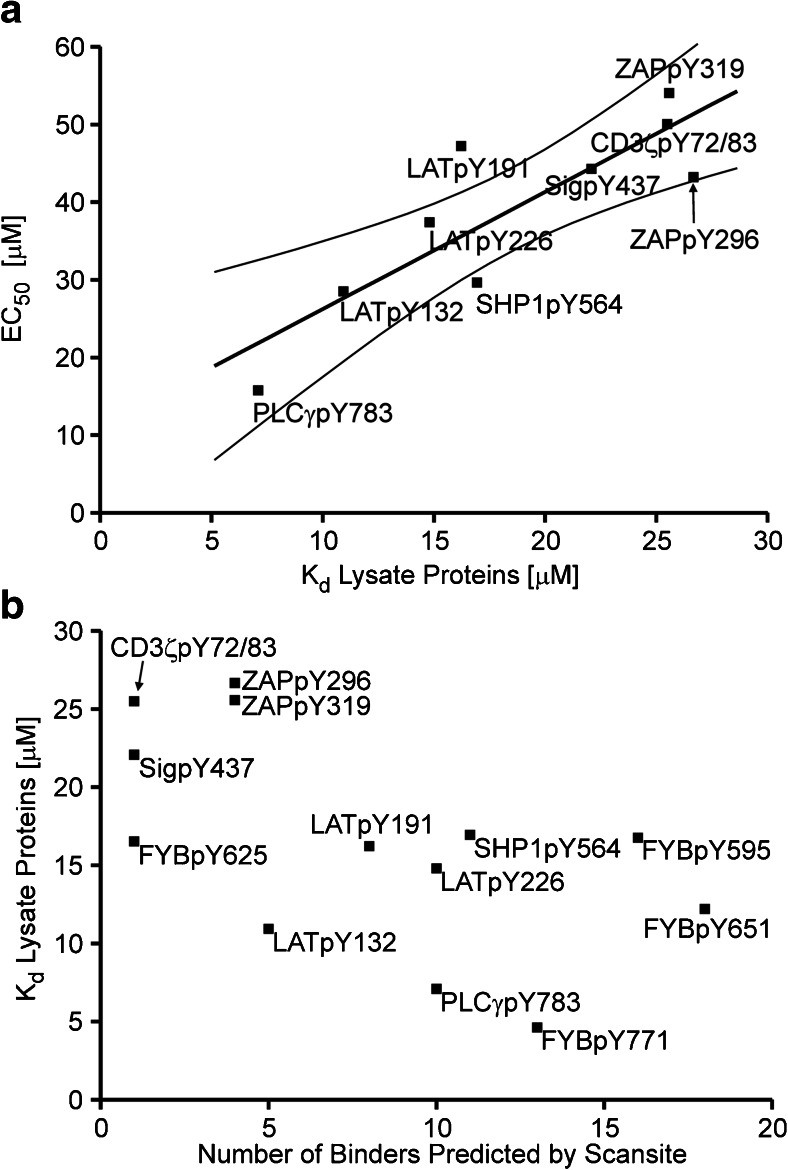
**a** Correlation of EC_50_ and *K*_d_ values of lysate proteins. The *K*_d_ values for the lysate proteins were determined using the Cheng-Prusoff equation. Next to the linear fit, a confidence interval of 0.95 is shown. **b** Plot of the number of binders predicted for the peptide sequences by the online resource Scansite versus the binding affinities of proteins in cell lysate

The competition experiments above provided information on the activity of a peptide as an SH2 domain-binding motif for the entire proteome that did not require any a priori knowledge on potential binders. However, strong competition could be a function of a relatively weaker binding of the α-pY antibody, a strong binding of the lysate or both. In order to correct the EC_50_ values for the binding characteristics of the α-pY antibody, the Cheng-Prusoff equation (Eq. 4) was applied for calculation of *K*_d_ values of the lysate proteins. Since for the binding of the antibody to the individual peptides the *K*_d_ values showed only little variation, there was a strong positive correlation of EC_50_ and *K*_d_ values (Fig. 3a).

Outliers of this correlation were SHP1pY564 and LATpY191. For SHP1pY564 and also for ZAPpY296, which were at the edge of the confidence interval, the α-pY antibody showed a relatively low affinity leading to a low EC_50_ value in the competition experiments with cell lysate and thus to an overestimation of the affinity by potential binders in the lysate. This is why these data showed a shift towards the *x*-axis. LATpY191, on the contrary, displayed a high affinity for the α-pY antibody (see also example in Fig. 2a), which caused an underestimation of the general affinity, reflected by a relatively high EC_50_. The conversion of EC_50_ values in *K*_d_ values accounts for differences in EC_50_ values resulting from different affinities of the antibody, thereby providing corrected information for the potential of the peptides for the binding to lysate proteins.

In addition, strong competition by the lysate could be a result of either high-affinity binding of individual proteins or of binding of several proteins. In order to address this question, the internet resource Scansite was used to predict the number of potential interactors. Scansite is based on results from oriented peptide library and phage display experiments and finds short protein sequence motifs that interact with modular signalling domains [18].

The number of binders as predicted by Scansite was plotted against the *K*_d_ values of the lysate proteins. For this examination, we also included four peptides derived from the adaptor protein FYB/ADAP from another experimental series. For these peptides, binders had been identified using mass spectrometry-based approaches [19] and the functional significance of individual phosphotyrosine residues had been addressed using a knock-in approach [20]. FYB/ADAP couples TCR stimulation to the activation of integrins by mediating increased integrin avidity [21]. Like other adaptor proteins, FYB/ADAP is phosphorylated on multiple sites and as a consequence involved in complex formation with several proteins [22]. In our competition experiments, all FYB-derived peptides had shown binding to the cellular proteome (see ESM, Fig. S1).

Remarkably, there was a clear correlation between the dissociation constants of the lysate proteins and the number of binders predicted by Scansite (Fig. 3b). A higher affinity for the cellular proteome correlated with a high number of predicted binders. This finding suggests that the collective affinity of a cellular proteome to a binding motif is, for a major part, rather determined by the number of binders than by the expression level and/or affinity of individual interactors.

The data on the FYB peptides was further validated by comparison to the recently published mass spectrometry results and functional analyses. In the microarray experiments, FYBpY771 showed the highest affinity of all four FYB peptides in line with data obtained by ^18^O labelling that had demonstrated that this site had the largest number of binding partners [19]. Moreover, mutation of this residue for phenylalanine had the strongest impact on T cell activation [20].

### Use of protein domains as pan-specific binders

For many peptide motifs, a pan-specific binder such as the α-pY antibody will not be available. In such cases, a protein domain with a broad-band binding specificity may serve as a surrogate. In order to explore this option for the phosphotyrosine-containing motifs, we conducted analogous competition experiments with the recombinant SH2 domain of the adaptor protein GRB2 [23, 24]. Again, we took advantage of the Cheng-Prusoff equation, using EC_50_ values obtained for titrations of GRB2-SH2 binding with cell lysate and *K*_d_ values of the recombinant GRB2-SH2 protein domain that were described in our recently published manuscript [14].

**Fig. 4:**
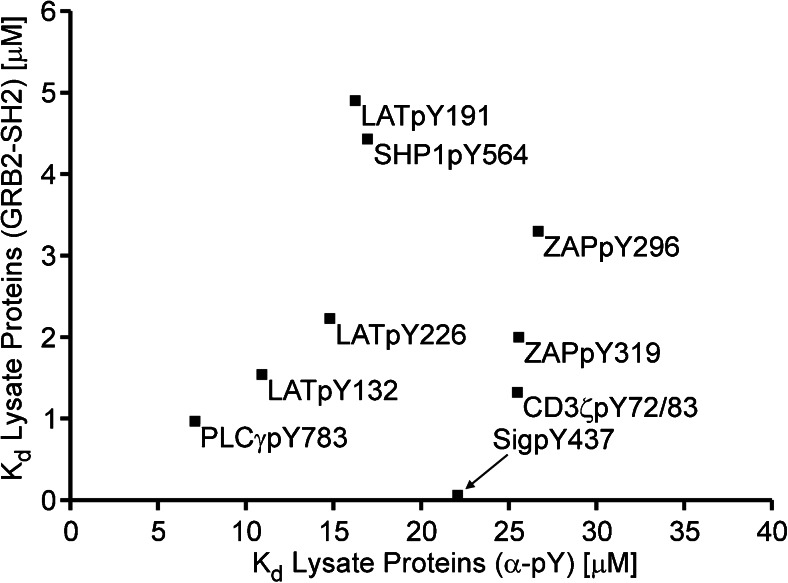
Plot of dissociation constants of endogenous binders in cell lysate derived from experiments with GRB2-SH2 versus the *K*_d_ of the cellular proteome derived from experiments with α-pY antibody. Data points represent mean values of six independent experiments

In analogy to the α-phosphotyrosine antibody, the titration of the recombinant GRB2-SH2 domain with cell lysates provides an approach for obtaining general information on the activity of peptide motifs as binders for the total cellular proteome. However, the GRB2-SH2 domain by itself possesses a clear preference for certain motifs, which may impose a restriction for the use of a protein domain as a reference binder. In order to assess the relevance of this restriction, we compared the *K*_d_ derived from the competition experiments with GRB2-SH2 and cell lysate with the *K*_d_ obtained from the experiments with the α-pY and cell lysate (Fig. 4).

The plot of these *K*_d_ values showed a good correlation for the motifs PLCγpY783, LATpY132, LATpY226 and ZAPpY296 for which GRB2 is a good binder. In contrast, the data points for LATpY191 and SHP1pY564, and SigpY437, ZAPpY319 and CDζpY72/83 were above or below the linear correlation defined by the first four peptides. The LATpY191 motif has a strong affinity for the SH2 domain of GRB2 [25, 26] leading to a rather small competition on this motif by other binders in the cell lysate. The same holds true for SHP1pY564 [27]. SigpY437, in contrast, is a poor binder for which already low concentrations of lysate show full competition.

## Discussion

Here, we present a competition-based microarray-approach to provide information on the significance of interaction motifs for the cellular proteome. The analyses benefited from a consequent adaptation of biochemical protocols to the microarray format. Titrations yielded binding isotherms for a recombinant protein and an antibody. By application of the Cheng-Prusoff equation to competitive titrations with crude cell lysates, these could then be translated into *K*_d_ values of the total proteome for the peptides on the microarrays. Importantly, the resulting predictions of binding capacity correlated well with Scansite predictions and, for the FYB/ADAP peptides, with standard proteomics and functional data. These results underscore and extend the potential of peptide microarrays in parallel biochemical approaches [13, 16].

Nevertheless, the absolute binding constants obtained in the various experiments also give clear indications that the microarray experiments are operating far from equilibrium. Binding constants for the cellular proteome using the GRB2-SH2 domain as a competitor were up to 20 times smaller than those obtained with the anti-phosphotyrosine antibody as a competitor. While with the GRB2 domain-binding constants were lower than typically expected for SH2 domains, for the antibody, they were higher. Previously, a strong agreement between microarray-derived and SPR-derived *K*_d_ values was observed if microarrays were directly titrated with fluorescently labelled peptides instead of protein domains followed by indirect immunofluorescence [10, 11, 16]. Indirect immunofluorescence labelling requires several wash steps and periods of incubation. To prevent dissociation of bound proteins, wash steps and incubations with antibodies were conducted as short as possible. Owing to the non-equilibrium binding kinetics and dissociation introduced by any one of these steps, the exact nature of the observed differences for the approach with GRB2-SH2 and the anti-phosphotyrosine antibody observed in our case remains unknown. It has to be noted that mass transport limitation effects on our microarrays cannot be ruled out. When using high affinity, high molecular weight binders, binding kinetics can rapidly become diffusion limited [6, 28, 29]. Thus, in this respect, we consider perfusion-based biosensing systems as powerful as our microarray-based approach. However, very clearly, the microarray operates at much lower sample consumption. Making larger amounts of cell lysates would be a limiting factor for a biosensing system.

For the GRB2-SH2 domain, the results demonstrate the general feasibility of the use of protein domains as reference ligands for the profiling of interaction motifs. However, the Cheng-Prusoff equation did not correct for major differences in the binding affinity of the reference binder, which may most likely also be attributed to the lack of equilibrium conditions. Therefore, the data has to be critically evaluated against the affinity of the pan-specific binder for the interaction motif.

In summary, the results presented in this research demonstrate the capacity of peptide microarrays to provide information on the significance of individual peptide motifs as protein binders for the total proteome. In contrast to previous approaches, this protocol does not require fluorescent labelling of the proteome [30–32]. Instead, detection of the reference binder is required. Even though there is no discrimination between an individual peptide binding a certain protein with very high affinity or several proteins with intermediate affinity, our comparison with the number of predicted binders by Scansite suggests that a strong interaction of the lysate for the binding motifs examined here is a result of many interactors. This information is highly valuable for a general assessment of the significance of a peptide/protein domain as an interaction motif.

## Electronic supplementary material

Below is the link to the electronic supplementary material.

ESM 1 (PDF 1.52 mb)
