# Supplementary material for: A microarray-based approach to evaluate the functional significance of protein-binding motifs
Source: Anal Bioanal Chem. 2016 Feb 18;408:3177–84. doi: 10.1007/s00216-016-9382-6 (PMC4830892; doi:10.1007/s00216-016-9382-6)
Supplement: Supplementary file 1 — (PDF 1.52 mb) [file 216_2016_9382_MOESM1_ESM.pdf]

Analytical and Bioanalytical Chemistry

Electronic Supplementary Material

A microarray-based approach to evaluate the functional significance of protein binding motifs

Michael D. Sinzinger, Yi-Da Chung, Merel J.W. Adjobo-Hermans, Roland Brock

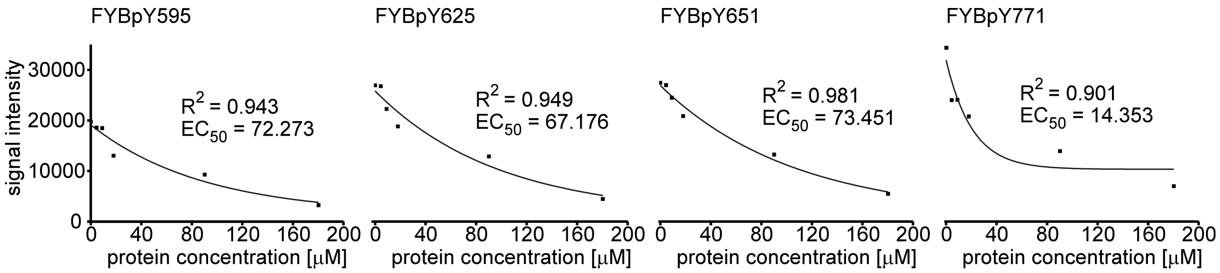

Figure S1 Examples for binding curves for the competition by cell lysate of Jurkat cells against a α-pY antibody on peptides derived from the adapter protein FYB. Competitions were performed with a fixed concentration of 0.33 nM α-pY and increasing concentrations of cell lysate. The displayed competition curves refer to the peptides mentioned above

Table S1 Arrayed peptides. Peptide designations are derived from the respective protein containing the peptide sequence; for phosphotyrosine (pY) peptides the number refers to the position of the pY residue within the protein. For proline-rich peptides, the number designates the first residue in the peptide

Peptide	Sequence	binding motif for domain	Source (UniProt ID)	Interacting partners (UniProt ID)
**ZAPpY296**	CIDTLNSDG(pY)TPEPARIT-NH2 [1]	PTB	ZAP-70 284-300 (P43403)	CBL (P22681)
**LATpY191**	CEASLDGSRE(pY)VNVSQEL-NH2 [2,3]	SH2	LAT 181-198 (O43561)	VAV (P15498), GADS (O75791), GRB2 (P62993), PLCγ1 (P19174)
**WAS334**	CGNKGRSGPLPPVPLGIA-NH2	SH3	hWASP 334-350 P42768	NCK (P16333)
**LATpY132**	CEDEDDYHNPG(pY)LVVLPDSTP-NH2 [4,2]	SH2	LAT 121-141 (O43561)	PLCγ1 (P19174)

**SigpY437**	CGEEREIQ(pY)APLSFHKG-NH2 [5]	SH2	SIGLEC-7 430-445 (Q9Y286)	SHPTP1 (P29350), SHPTP2 (Q06124)
**PLCγpY783**	CEGRNPGF(pY)VEANPMPT-NH2 [6]	SH2	PLCg1 775-791 (P19174)	PLCg1 intramolecular (P19174)
**ZAPpY319**	CVYESP(pY)SDPEELKD-NH2 [7]	SH2	ZAP70 314-327 (P43403)	LCK (P06239)
**LATpY226**	CEVEEEGAPD(pY)ENLQELN-NH2 [8]	SH2	LAT 217-233 (O43561)	GADS (O75791), GRB2 (P62993), VAV (P15498)
**SHP1pY564**	CSKHKEDV(pY)ENLHTKNK-NH2 [9]	SH2	SHP1 557-572 (P29350)	LCK (P06239)
**CD3ζpY72/83**	C-Ahx-NQL(pY)NELNLGRREE(pY)DVL-NH2 [10]	Tandem-SH2	CD3ζ ITAM1 69-86 (P24161)	ZAP70 (P43403)
**SLP179**	CSGKTPQQPPVPPQRPMAAL-NH2 [11]	SH3	SLP76 179-197 (Q13094)	PLCg1 (P19174)
**PAK6**	CLDIQDKPPAPPMRNT-NH2 [12]	SH3	PAK1 6-20 (Q13153)	NCK (P16333)
**SLP228**	CAKLPAPSIDRSTKPPLDRS-CONH2 [13]	SH3	SLP76 228-246 (Q13094)	GADS (O75791)
**PI3K84**	CPTPKPRPPRPLPVAPGSSKT-NH2 [14]	SH3	PI3Kp85 84-104 (P27986)	FYN (P06241)
**Fyb pY595**	CEDDQEV(pY)DDVAEQD-NH2 [15]	SH2	Fyb 589-602 (O15117)	FYN (P06241), SLP76 (Q13094)
**Fyb pY625**	CDDDI(pY)DGIEEED-NH2 [15]	SH2	Fyb 621-632 (O15117)	FYN (P06241),
**Fyb pY651**	CLDMGDEV(pY)DDVDTSDF-NH2 [15]	SH2	Fyb 644-659 (O15117)	SLP76 (Q13094)

**FYB pY771**	CNDGEI(pY)DDIADG [16]	SH2	Fyb, 767-777 (O15117)	c-Src (P12931)

References

1. Lupher ML, Songyang Z, Shoelson SE, Cantley LC, Band H (1997) The Cbl phosphotyrosine-binding domain selects a D(N/D)XpY motif and binds to the Tyr292 negative regulatory phosphorylation site of ZAP-70. J Biol Chem 272 (52):33140-33144

2. Paz PE, Wang S, Clarke H, Lu X, Stokoe D, Abo A (2001) Mapping the Zap-70 phosphorylation sites on LAT (linker for activation of T cells) required for recruitment and activation of signalling proteins in T cells. Biochem J 356 (Pt 2):461-471

3. Zhu M, Janssen E, Zhang W (2003) Minimal requirement of tyrosine residues of linker for activation of T cells in TCR signaling and thymocyte development. J Immunol 170 (1):325-333

4. Zhang W, Trible RP, Zhu M, Liu SK, McGlade CJ, Samelson LE (2000) Association of Grb2, Gads, and phospholipase C-gamma 1 with phosphorylated LAT tyrosine residues. Effect of LAT tyrosine mutations on T cell angigen receptor-mediated signaling. J Biol Chem 275 (30):23355-23361. doi:10.1074/jbc.M000404200

5. Nicoll G, Ni J, Liu D, Klenerman P, Munday J, Dubock S, Mattei MG, Crocker PR (1999) Identification and characterization of a novel siglec, siglec-7, expressed by human natural killer cells and monocytes. J Biol Chem 274 (48):34089-34095

6. Poulin B, Sekiya F, Rhee SG (2005) Intramolecular interaction between phosphorylated tyrosine-783 and the C-terminal Src homology 2 domain activates phospholipase C-gamma1. Proc Natl Acad Sci USA 102 (12):4276-4281. doi:10.1073/pnas.0409590102

7. Pelosi M, Di Bartolo V, Mounier V, Mège D, Pascussi JM, Dufour E, Blondel A, Acuto O (1999) Tyrosine 319 in the interdomain B of ZAP-70 is a binding site for the Src homology 2 domain of Lck. J Biol Chem 274 (20):14229-14237

8. Lin J, Weiss A (2001) Identification of the minimal tyrosine residues required for linker for activation of T cell function. J Biol Chem 276 (31):29588-29595. doi:10.1074/jbc.M102221200

9. Cuevas B, Lu Y, Watt S, Kumar R, Zhang J, Siminovitch KA, Mills GB (1999) SHP-1 regulates Lck-induced phosphatidylinositol 3-kinase phosphorylation and activity. J Biol Chem 274 (39):27583-27589

10. Goda S, Quale AC, Woods ML, Felthauser A, Shimizu Y (2004) Control of TCR-mediated activation of beta 1 integrins by the ZAP-70 tyrosine kinase interdomain B region and the linker for activation of T cells adapter protein. J Immunol 172 (9):5379-5387

11. Deng L, Velikovsky CA, Swaminathan CP, Cho S, Mariuzza RA (2005) Structural basis for recognition of the T cell adaptor protein SLP-76 by the SH3 domain of phospholipase Cgamma1. J Mol Biol 352 (1):1-10. doi:10.1016/j.jmb.2005.06.072

12. Bokoch GM, Wang Y, Bohl BP, Sells MA, Quilliam LA, Knaus UG (1996) Interaction of the Nck adapter protein with p21-activated kinase (PAK1). J Biol Chem 271 (42):25746-25749

13. Harkiolaki M, Lewitzky M, Gilbert RJC, Jones EY, Bourette RP, Mouchiroud G, Sondermann H, Moarefi I, Feller SM (2003) Structural basis for SH3 domain-mediated high-affinity binding between Mona/Gads and SLP-76. EMBO J 22 (11):2571-2582. doi:10.1093/emboj/cdg258

14. Mak P, He Z, Kurosaki T (1996) Identification of amino acid residues required for a specific interaction between Src-tyrosine kinase and proline-rich region of phosphatidylinositol-3' kinase. FEBS Lett 397 (2-3):183-185

15. Geng L, Rudd C (2001) Adaptor ADAP (adhesion- and degranulation-promoting adaptor protein) regulates beta1 integrin clustering on mast cells. Biochem Biophys Res Commun 289 (5):1135-1140. doi:10.1006/bbrc.2001.6117

16. Koga S, Yogo K, Yoshikawa K, Samori H, Goto M, Uchida T, Ishida N, Takeya T (2005) Physical and functional association of c-Src and adhesion and degranulation promoting adaptor protein (ADAP) in osteoclastogenesis in vitro. J Biol Chem 280 (36):31564-31571. doi:10.1074/jbc.M502703200
